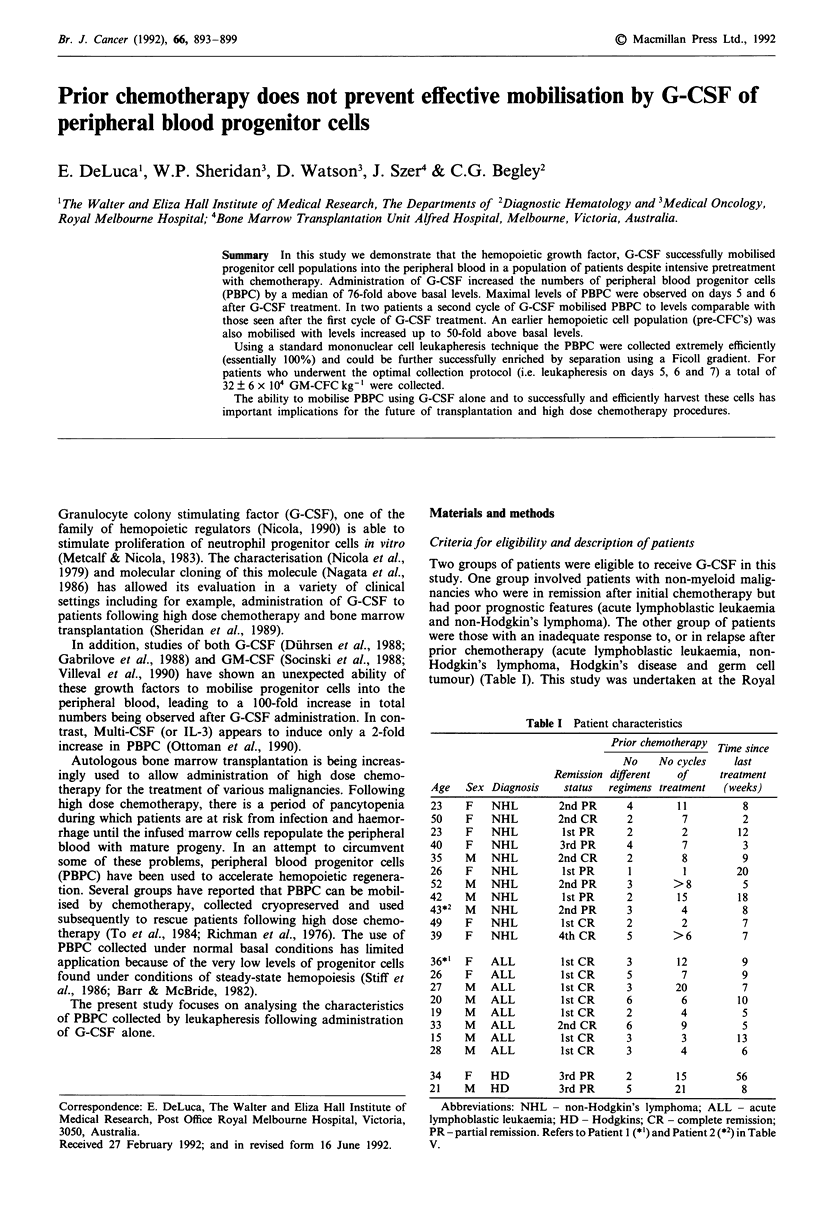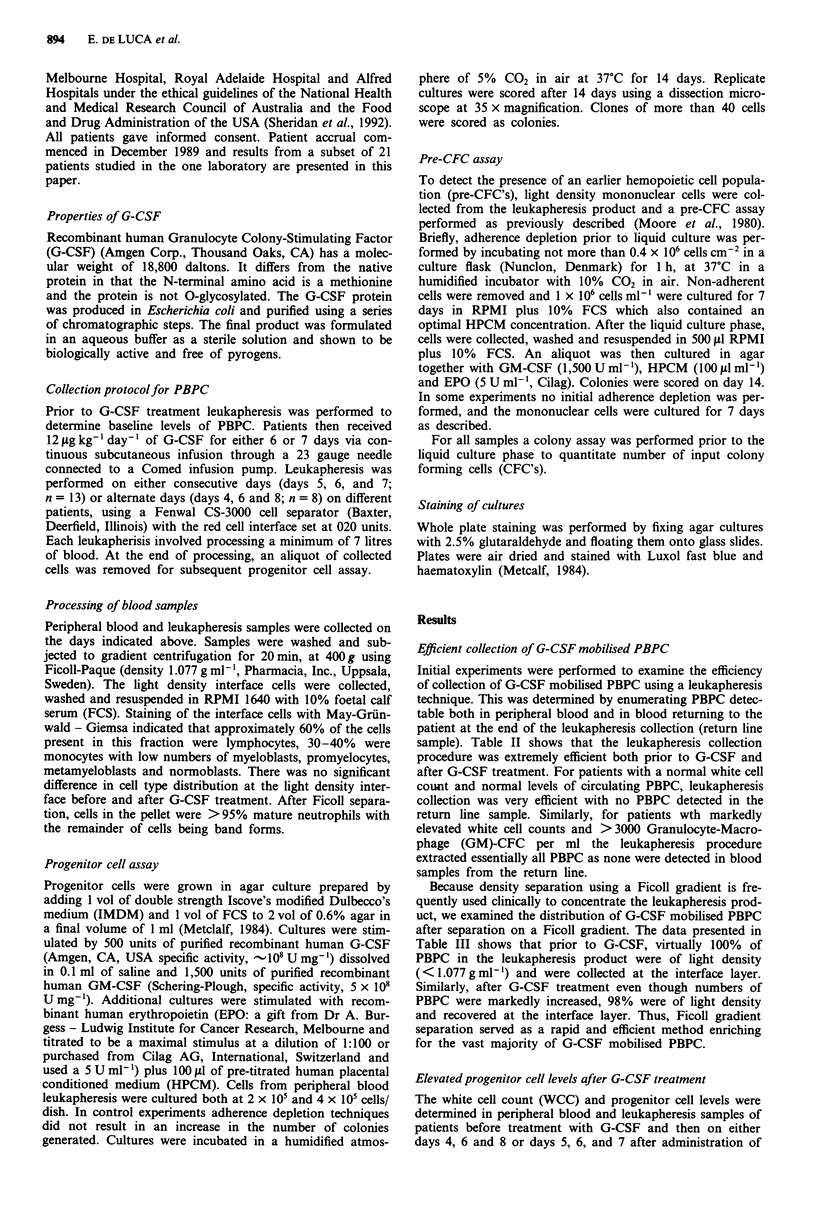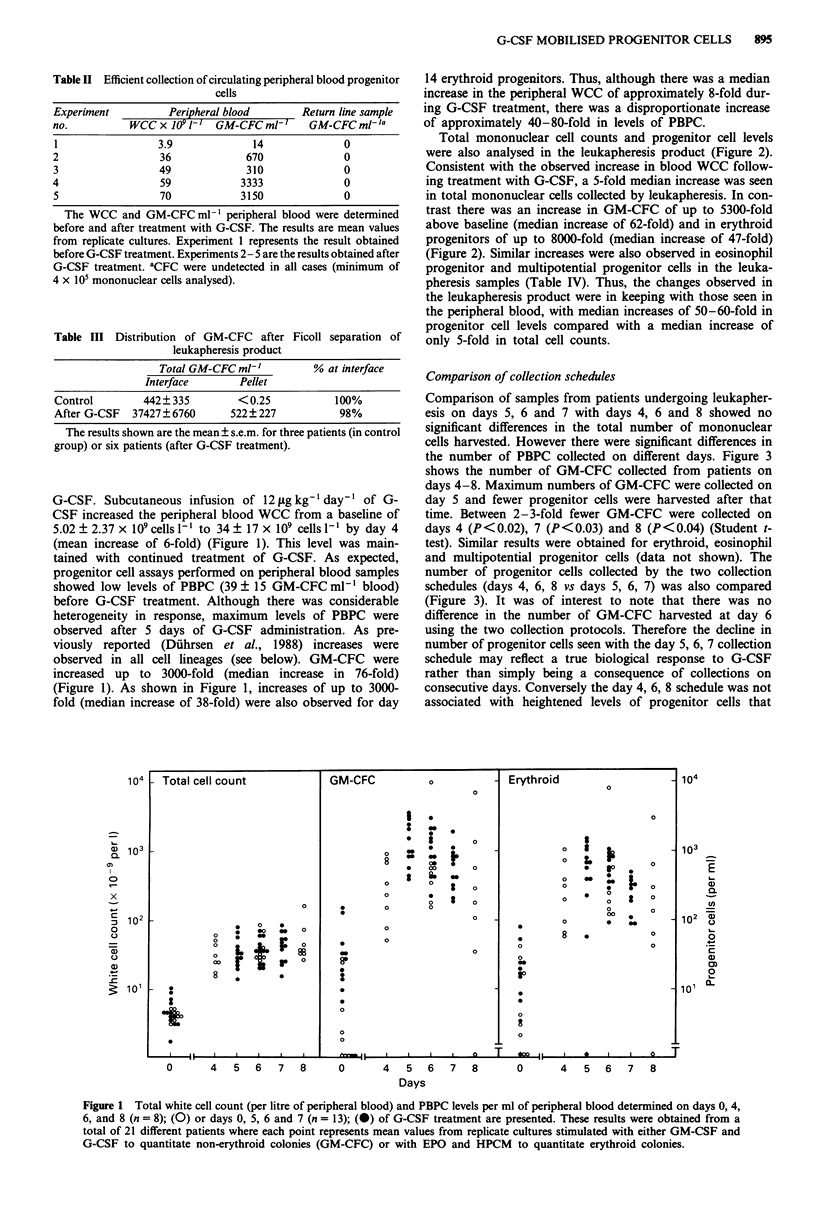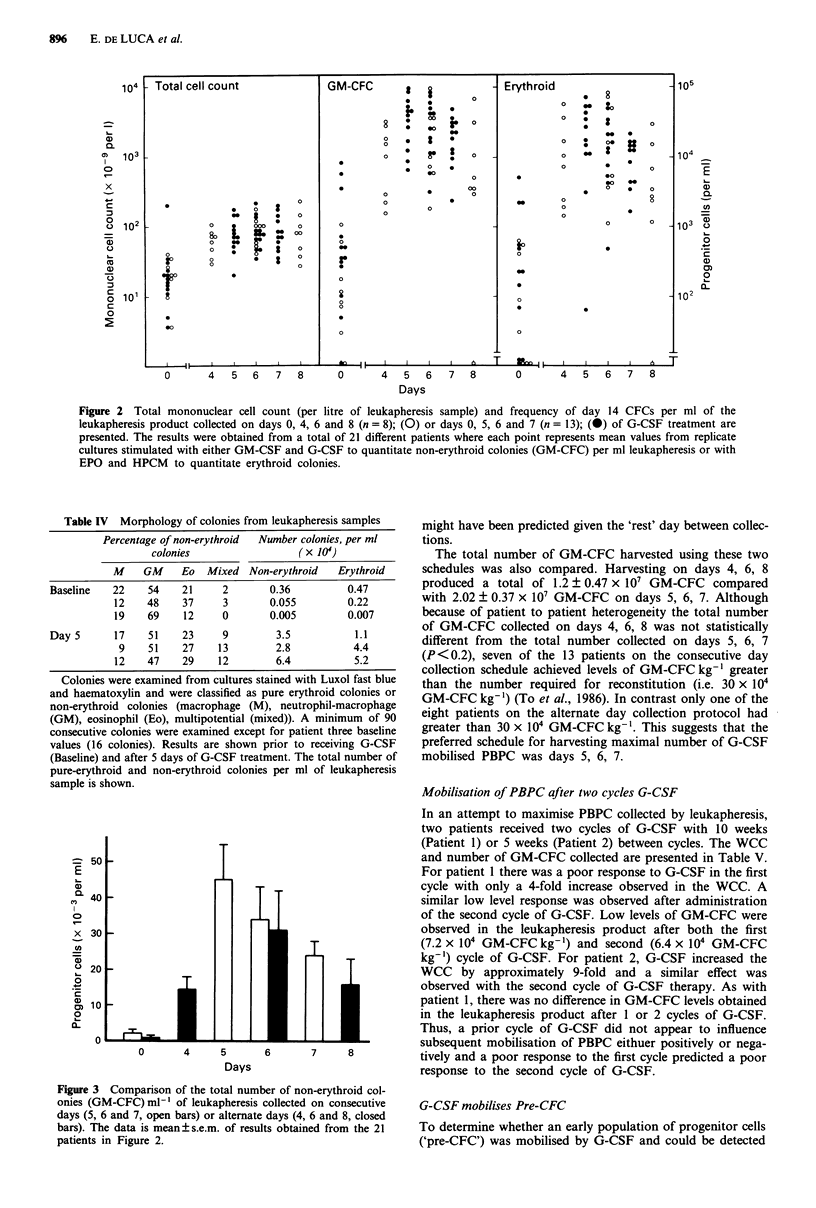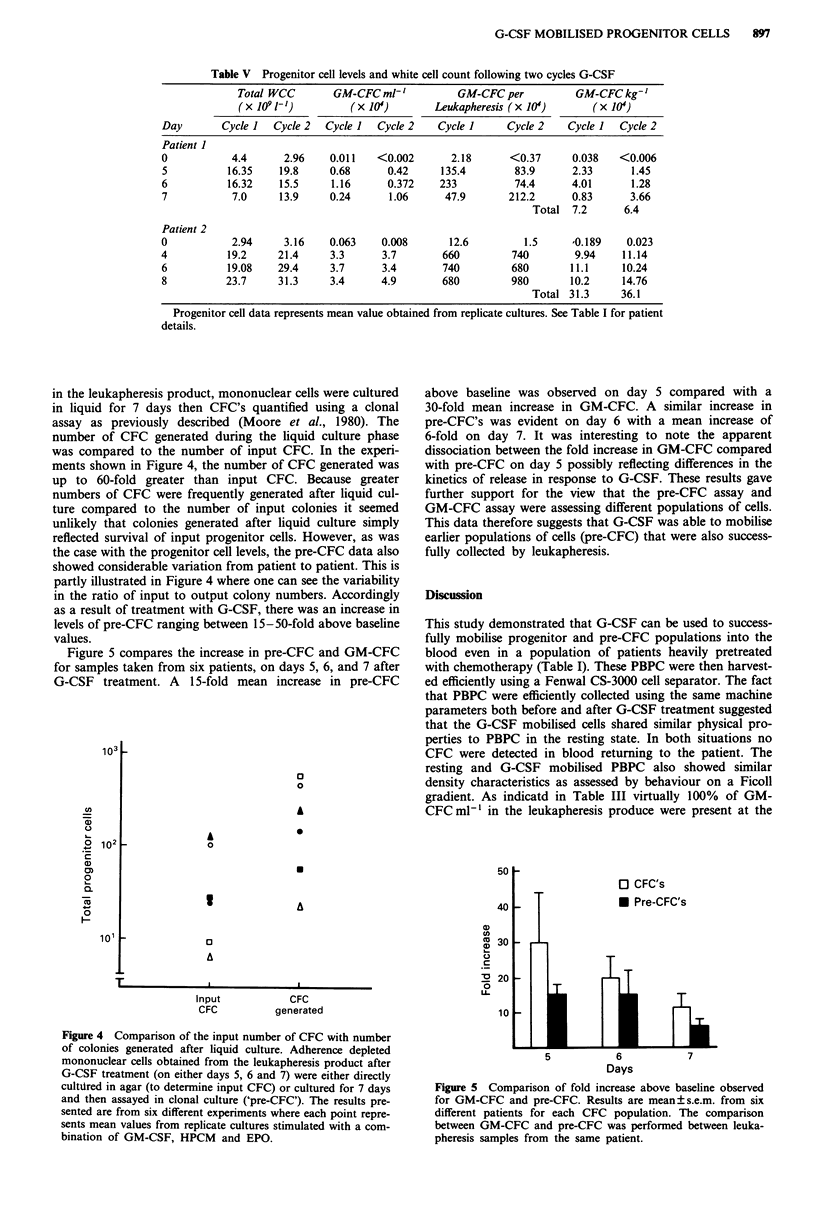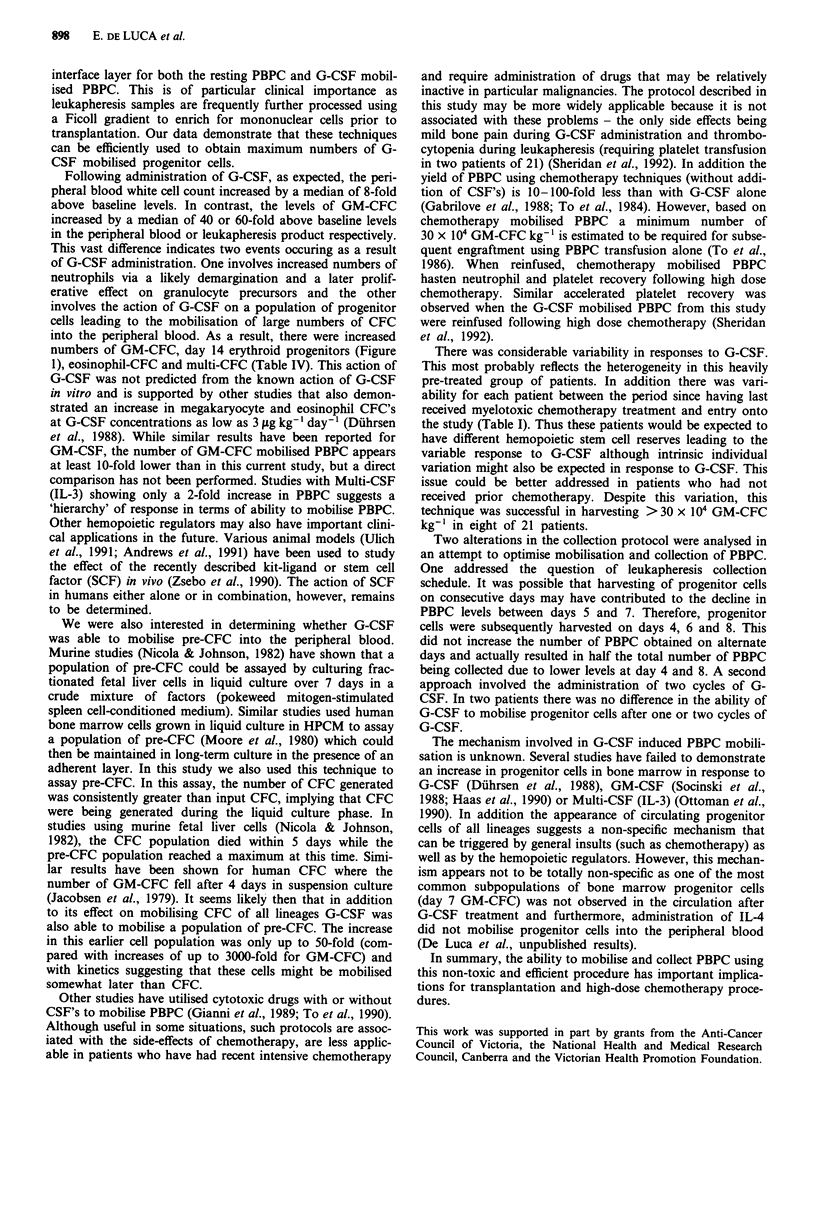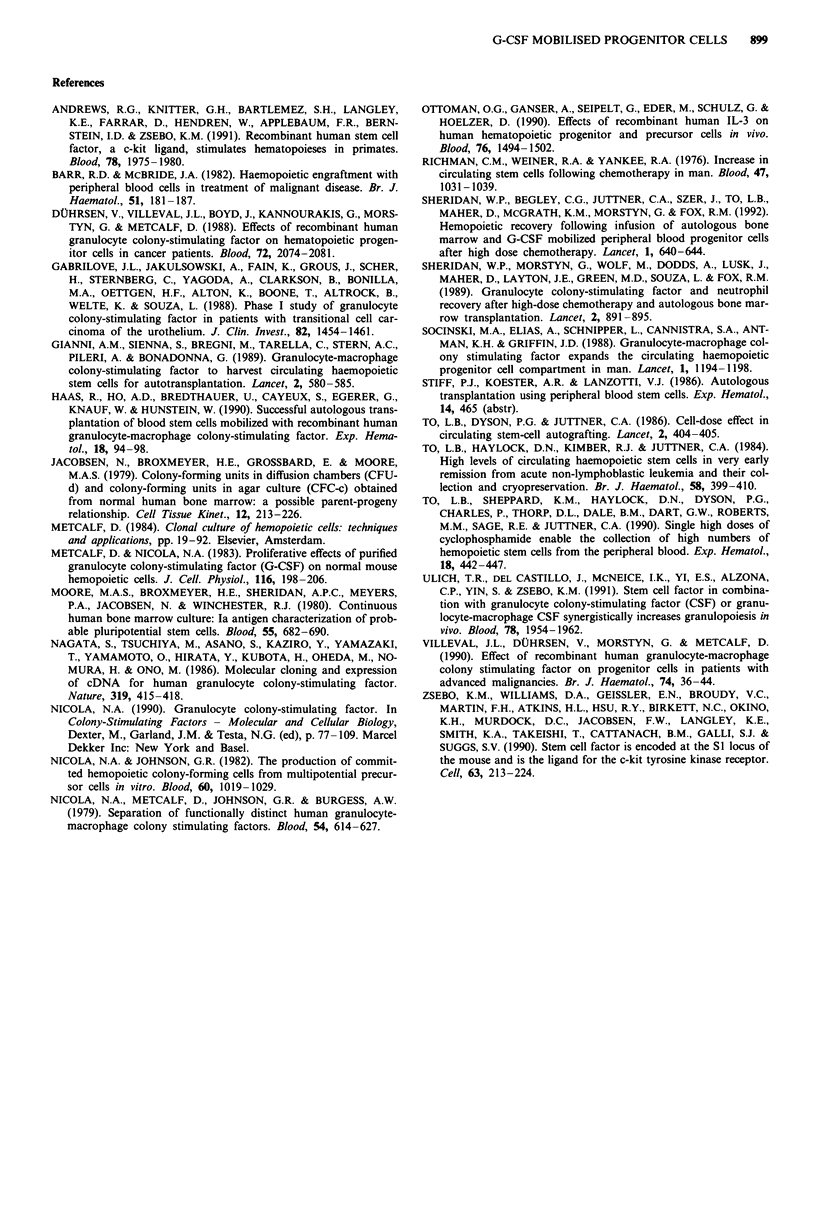# Prior chemotherapy does not prevent effective mobilisation by G-CSF of peripheral blood progenitor cells.

**DOI:** 10.1038/bjc.1992.381

**Published:** 1992-11

**Authors:** E. DeLuca, W. P. Sheridan, D. Watson, J. Szer, C. G. Begley

**Affiliations:** Walter and Eliza Hall Institute of Medical Research, Royal Melbourne Hospital, Victoria, Australia.

## Abstract

In this study we demonstrate that the hemopoietic growth factor, G-CSF successfully mobilised progenitor cell populations into the peripheral blood in a population of patients despite intensive pretreatment with chemotherapy. Administration of G-CSF increased the numbers of peripheral blood progenitor cells (PBPC) by a median of 76-fold above basal levels. Maximal levels of PBPC were observed on days 5 and 6 after G-CSF treatment. In two patients a second cycle of G-CSF mobilised PBPC to levels comparable with those seen after the first cycle of G-CSF treatment. An earlier hemopoietic cell population (pre-CFC's) was also mobilised with levels increased up to 50-fold above basal levels. Using a standard mononuclear cell leukapheresis technique the PBPC were collected extremely efficiently (essentially 100%) and could be further successfully enriched by separation using a Ficoll gradient. For patients who underwent the optimal collection protocol (i.e. leukapheresis on days 5, 6 and 7) a total of 32 +/- 6 x 10(4) GM-CFC kg-1 were collected. The ability to mobilise PBPC using G-CSF alone and to successfully and efficiently harvest these cells has important implications for the future of transplantation and high dose chemotherapy procedures.


Br. J. Cancer (1992), 66, 893-899 © Macmillan Press Ltd., 1992

### Prior chemotherapy does not prevent effective mobilisation by G-CSF of peripheral blood progenitor cells

E. DeLuca[1], W.P. Sheridan[3], D. Watson[3], J. Szer[4] & C.G. Begley[2]

[1]*The Walter and Eliza Hall Institute of Medical Research, The Departments of* [2]*Diagnostic Hematology and* [3]*Medical Oncology, Royal Melbourne Hospital;* [4]*Bone Marrow Transplantation Unit Alfred Hospital, Melbourne, Victoria, Australia.*

**Summary** In this study we demonstrate that the hemopoietic growth factor, G-CSF successfully mobilised progenitor cell populations into the peripheral blood in a population of patients despite intensive pretreatment with chemotherapy. Administration of G-CSF increased the numbers of peripheral blood progenitor cells (PBPC) by a median of 76-fold above basal levels. Maximal levels of PBPC were observed on days 5 and 6 after G-CSF treatment. In two patients a second cycle of G-CSF mobilised PBPC to levels comparable with those seen after the first cycle of G-CSF treatment. An earlier hemopoietic cell population (pre-CFC's) was also mobilised with levels increased up to 50-fold above basal levels.

Using a standard mononuclear cell leukapheresis technique the PBPC were collected extremely efficiently (essentially 100%) and could be further successfully enriched by separation using a Ficoll gradient. For patients who underwent the optimal collection protocol (i.e. leukapheresis on days 5, 6 and 7) a total of $32 \pm 6 \times 10^4$ GM-CFC $kg^{-1}$ were collected.

The ability to mobilise PBPC using G-CSF alone and to successfully and efficiently harvest these cells has important implications for the future of transplantation and high dose chemotherapy procedures.

Granulocyte colony stimulating factor (G-CSF), one of the family of hemopoietic regulators (Nicola, 1990) is able to stimulate proliferation of neutrophil progenitor cells *in vitro* (Metcalf & Nicola, 1983). The characterisation (Nicola *et al.*, 1979) and molecular cloning of this molecule (Nagata *et al.*, 1986) has allowed its evaluation in a variety of clinical settings including for example, administration of G-CSF to patients following high dose chemotherapy and bone marrow transplantation (Sheridan *et al.*, 1989).

In addition, studies of both G-CSF (Dührsen *et al.*, 1988; Gabrilove *et al.*, 1988) and GM-CSF (Socinski *et al.*, 1988; Villeval *et al.*, 1990) have shown an unexpected ability of these growth factors to mobilise progenitor cells into the peripheral blood, leading to a 100-fold increase in total numbers being observed after G-CSF administration. In contrast, Multi-CSF (or IL-3) appears to induce only a 2-fold increase in PBPC (Ottoman *et al.*, 1990).

Autologous bone marrow transplantation is being increasingly used to allow administration of high dose chemotherapy for the treatment of various malignancies. Following high dose chemotherapy, there is a period of pancytopenia during which patients are at risk from infection and haemorrhage until the infused marrow cells repopulate the peripheral blood with mature progeny. In an attempt to circumvent some of these problems, peripheral blood progenitor cells (PBPC) have been used to accelerate hemopoietic regeneration. Several groups have reported that PBPC can be mobilised by chemotherapy, collected cryopreserved and used subsequently to rescue patients following high dose chemotherapy (To *et al.*, 1984; Richman *et al.*, 1976). The use of PBPC collected under normal basal conditions has limited application because of the very low levels of progenitor cells found under conditions of steady-state hemopoiesis (Stiff *et al.*, 1986; Barr & McBride, 1982).

The present study focuses on analysing the characteristics of PBPC collected by leukapheresis following administration of G-CSF alone.

Correspondence: E. DeLuca, The Walter and Eliza Hall Institute of Medical Research, Post Office Royal Melbourne Hospital, Victoria, 3050, Australia.
Received 27 February 1992; and in revised form 16 June 1992.

### Materials and methods

### *Criteria for eligibility and description of patients*

Two groups of patients were eligible to receive G-CSF in this study. One group involved patients with non-myeloid malignancies who were in remission after initial chemotherapy but had poor prognostic features (acute lymphoblastic leukaemia and non-Hodgkin's lymphoma). The other group of patients were those with an inadequate response to, or in relapse after prior chemotherapy (acute lymphoblastic leukaemia, non-Hodgkin's lymphoma, Hodgkin's disease and germ cell tumour) (Table I). This study was undertaken at the Royal

**Table I** Patient characteristics

| | | | | *Prior chemotherapy* | | |
|---|---|---|---|---|---|---|
| *Age* | *Sex* | *Diagnosis* | *Remission status* | *No different regimens* | *No cycles of treatment* | *Time since last treatment (weeks)* |
| 23 | F | NHL | 2nd PR | 4 | 11 | 8 |
| 50 | F | NHL | 2nd CR | 2 | 7 | 2 |
| 23 | F | NHL | 1st PR | 2 | 2 | 12 |
| 40 | F | NHL | 3rd PR | 4 | 7 | 3 |
| 35 | M | NHL | 2nd CR | 2 | 8 | 9 |
| 26 | F | NHL | 1st PR | 1 | 1 | 20 |
| 52 | M | NHL | 2nd PR | 3 | >8 | 5 |
| 42 | M | NHL | 1st PR | 2 | 15 | 18 |
| 43*[2] | M | NHL | 2nd PR | 3 | 4 | 8 |
| 49 | F | NHL | 1st CR | 2 | 2 | 7 |
| 39 | F | NHL | 4th CR | 5 | >6 | 7 |
| 36*[1] | F | ALL | 1st CR | 3 | 12 | 9 |
| 26 | F | ALL | 1st CR | 5 | 7 | 9 |
| 27 | M | ALL | 1st CR | 3 | 20 | 7 |
| 20 | M | ALL | 1st CR | 6 | 6 | 10 |
| 19 | M | ALL | 1st CR | 2 | 4 | 5 |
| 33 | M | ALL | 2nd CR | 6 | 9 | 5 |
| 15 | M | ALL | 1st CR | 3 | 3 | 13 |
| 28 | M | ALL | 1st CR | 3 | 4 | 6 |
| 34 | F | HD | 3rd PR | 2 | 15 | 56 |
| 21 | M | HD | 3rd PR | 5 | 21 | 8 |

Abbreviations: NHL – non-Hodgkin's lymphoma; ALL – acute lymphoblastic leukaemia; HD – Hodgkins; CR – complete remission; PR – partial remission. Refers to Patient 1 (*[1]) and Patient 2 (*[2]) in Table V.

Melbourne Hospital, Royal Adelaide Hospital and Alfred Hospitals under the ethical guidelines of the National Health and Medical Research Council of Australia and the Food and Drug Administration of the USA (Sheridan et al., 1992). All patients gave informed consent. Patient accrual commenced in December 1989 and results from a subset of 21 patients studied in the one laboratory are presented in this paper.

*Properties of G-CSF*

Recombinant human Granulocyte Colony-Stimulating Factor (G-CSF) (Amgen Corp., Thousand Oaks, CA) has a molecular weight of 18,800 daltons. It differs from the native protein in that the N-terminal amino acid is a methionine and the protein is not O-glycosylated. The G-CSF protein was produced in *Escherichia coli* and purified using a series of chromatographic steps. The final product was formulated in an aqueous buffer as a sterile solution and shown to be biologically active and free of pyrogens.

*Collection protocol for PBPC*

Prior to G-CSF treatment leukapheresis was performed to determine baseline levels of PBPC. Patients then received $12\,\mu g\,kg^{-1}\,day^{-1}$ of G-CSF for either 6 or 7 days via continuous subcutaneous infusion through a 23 gauge needle connected to a Comed infusion pump. Leukapheresis was performed on either consecutive days (days 5, 6, and 7; $n = 13$) or alternate days (days 4, 6 and 8; $n = 8$) on different patients, using a Fenwal CS-3000 cell separator (Baxter, Deerfield, Illinois) with the red cell interface set at 020 units. Each leukapheresis involved processing a minimum of 7 litres of blood. At the end of processing, an aliquot of collected cells was removed for subsequent progenitor cell assay.

*Processing of blood samples*

Peripheral blood and leukapheresis samples were collected on the days indicated above. Samples were washed and subjected to gradient centrifugation for 20 min, at 400 *g* using Ficoll-Paque (density $1.077\,g\,ml^{-1}$, Pharmacia, Inc., Uppsala, Sweden). The light density interface cells were collected, washed and resuspended in RPMI 1640 with 10% foetal calf serum (FCS). Staining of the interface cells with May-Grünwald - Giemsa indicated that approximately 60% of the cells present in this fraction were lymphocytes, 30–40% were monocytes with low numbers of myeloblasts, promyelocytes, metamyeloblasts and normoblasts. There was no significant difference in cell type distribution at the light density interface before and after G-CSF treatment. After Ficoll separation, cells in the pellet were $>95\%$ mature neutrophils with the remainder of cells being band forms.

*Progenitor cell assay*

Progenitor cells were grown in agar culture prepared by adding 1 vol of double strength Iscove's modified Dulbecco's medium (IMDM) and 1 vol of FCS to 2 vol of 0.6% agar in a final volume of 1 ml (Metclaf, 1984). Cultures were stimulated by 500 units of purified recombinant human G-CSF (Amgen, CA, USA specific activity, $\sim 10^8\,U\,mg^{-1}$) dissolved in 0.1 ml of saline and 1,500 units of purified recombinant human GM-CSF (Schering-Plough, specific activity, $5 \times 10^8\,U\,mg^{-1}$). Additional cultures were stimulated with recombinant human erythropoietin (EPO: a gift from Dr A. Burgess - Ludwig Institute for Cancer Research, Melbourne and titrated to be a maximal stimulus at a dilution of 1:100 or purchased from Cilag AG, International, Switzerland and used a $5\,U\,ml^{-1}$) plus $100\,\mu l$ of pre-titrated human placental conditioned medium (HPCM). Cells from peripheral blood leukapheresis were cultured both at $2 \times 10^5$ and $4 \times 10^5$ cells/dish. In control experiments adherence depletion techniques did not result in an increase in the number of colonies generated. Cultures were incubated in a humidified atmosphere of 5% $CO_2$ in air at 37°C for 14 days. Replicate cultures were scored after 14 days using a dissection microscope at 35 × magnification. Clones of more than 40 cells were scored as colonies.

*Pre-CFC assay*

To detect the presence of an earlier hemopoietic cell population (pre-CFC's), light density mononuclear cells were collected from the leukapheresis product and a pre-CFC assay performed as previously described (Moore et al., 1980). Briefly, adherence depletion prior to liquid culture was performed by incubating not more than $0.4 \times 10^6$ cells $cm^{-2}$ in a culture flask (Nunclon, Denmark) for 1 h, at 37°C in a humidified incubator with 10% $CO_2$ in air. Non-adherent cells were removed and $1 \times 10^6$ cells $ml^{-1}$ were cultured for 7 days in RPMI plus 10% FCS which also contained an optimal HPCM concentration. After the liquid culture phase, cells were collected, washed and resuspended in 500 $\mu l$ RPMI plus 10% FCS. An aliquot was then cultured in agar together with GM-CSF ($1,500\,U\,ml^{-1}$), HPCM ($100\,\mu l\,ml^{-1}$) and EPO ($5\,U\,ml^{-1}$, Cilag). Colonies were scored on day 14. In some experiments no initial adherence depletion was performed, and the mononuclear cells were cultured for 7 days as described.

For all samples a colony assay was performed prior to the liquid culture phase to quantitate number of input colony forming cells (CFC's).

*Staining of cultures*

Whole plate staining was performed by fixing agar cultures with 2.5% glutaraldehyde and floating them onto glass slides. Plates were air dried and stained with Luxol fast blue and haematoxylin (Metcalf, 1984).

### Results

*Efficient collection of G-CSF mobilised PBPC*

Initial experiments were performed to examine the efficiency of collection of G-CSF mobilised PBPC using a leukapheresis technique. This was determined by enumerating PBPC detectable both in peripheral blood and in blood returning to the patient at the end of the leukapheresis collection (return line sample). Table II shows that the leukapheresis collection procedure was extremely efficient both prior to G-CSF and after G-CSF treatment. For patients with a normal white cell count and normal levels of circulating PBPC, leukapheresis collection was very efficient with no PBPC detected in the return line sample. Similarly, for patients wth markedly elevated white cell counts and $>3000$ Granulocyte-Macrophage (GM)-CFC per ml the leukapheresis procedure extracted essentially all PBPC as none were detected in blood samples from the return line.

Because density separation using a Ficoll gradient is frequently used clinically to concentrate the leukapheresis product, we examined the distribution of G-CSF mobilised PBPC after separation on a Ficoll gradient. The data presented in Table III shows that prior to G-CSF, virtually 100% of PBPC in the leukapheresis product were of light density ($<1.077\,g\,ml^{-1}$) and were collected at the interface layer. Similarly, after G-CSF treatment even though numbers of PBPC were markedly increased, 98% were of light density and recovered at the interface layer. Thus, Ficoll gradient separation served as a rapid and efficient method enriching for the vast majority of G-CSF mobilised PBPC.

*Elevated progenitor cell levels after G-CSF treatment*

The white cell count (WCC) and progenitor cell levels were determined in peripheral blood and leukapheresis samples of patients before treatment with G-CSF and then on either days 4, 6 and 8 or days 5, 6, and 7 after administration of

**Table II** Efficient collection of circulating peripheral blood progenitor cells

| *Experiment no.* | *Peripheral blood WCC × $10^9$ $l^{-1}$* | *Peripheral blood GM-CFC $ml^{-1}$* | *Return line sample GM-CFC $ml^{-1a}$* |
|---|---|---|---|
| 1 | 3.9 | 14 | 0 |
| 2 | 36 | 670 | 0 |
| 3 | 49 | 310 | 0 |
| 4 | 59 | 3333 | 0 |
| 5 | 70 | 3150 | 0 |

The WCC and GM-CFC $ml^{-1}$ peripheral blood were determined before and after treatment with G-CSF. The results are mean values from replicate cultures. Experiment 1 represents the result obtained before G-CSF treatment. Experiments 2–5 are the results obtained after G-CSF treatment. [a]CFC were undetected in all cases (minimum of $4 \times 10^5$ mononuclear cells analysed).

**Table III** Distribution of GM-CFC after Ficoll separation of leukapheresis product

| | *Total GM-CFC $ml^{-1}$ Interface* | *Total GM-CFC $ml^{-1}$ Pellet* | *% at interface* |
|---|---|---|---|
| Control | 442±335 | <0.25 | 100% |
| After G-CSF | 37427±6760 | 522±227 | 98% |

The results shown are the mean ± s.e.m. for three patients (in control group) or six patients (after G-CSF treatment).

G-CSF. Subcutaneous infusion of 12 μg $kg^{-1}$ $day^{-1}$ of G-CSF increased the peripheral blood WCC from a baseline of $5.02 \pm 2.37 \times 10^9$ cells $l^{-1}$ to $34 \pm 17 \times 10^9$ cells $l^{-1}$ by day 4 (mean increase of 6-fold) (Figure 1). This level was maintained with continued treatment of G-CSF. As expected, progenitor cell assays performed on peripheral blood samples showed low levels of PBPC ($39 \pm 15$ GM-CFC $ml^{-1}$ blood) before G-CSF treatment. Although there was considerable heterogeneity in response, maximum levels of PBPC were observed after 5 days of G-CSF administration. As previously reported (Dührsen *et al.*, 1988) increases were observed in all cell lineages (see below). GM-CFC were increased up to 3000-fold (median increase in 76-fold) (Figure 1). As shown in Figure 1, increases of up to 3000-fold (median increase of 38-fold) were also observed for day 14 erythroid progenitors. Thus, although there was a median increase in the peripheral WCC of approximately 8-fold during G-CSF treatment, there was a disproportionate increase of approximately 40–80-fold in levels of PBPC.

Total mononuclear cell counts and progenitor cell levels were also analysed in the leukapheresis product (Figure 2). Consistent with the observed increase in blood WCC following treatment with G-CSF, a 5-fold median increase was seen in total mononuclear cells collected by leukapheresis. In contrast there was an increase in GM-CFC of up to 5300-fold above baseline (median increase of 62-fold) and in erythroid progenitors of up to 8000-fold (median increase of 47-fold) (Figure 2). Similar increases were also observed in eosinophil progenitor and multipotential progenitor cells in the leukapheresis samples (Table IV). Thus, the changes observed in the leukapheresis product were in keeping with those seen in the peripheral blood, with median increases of 50–60-fold in progenitor cell levels compared with a median increase of only 5-fold in total cell counts.

*Comparison of collection schedules*

Comparison of samples from patients undergoing leukapheresis on days 5, 6 and 7 with days 4, 6 and 8 showed no significant differences in the total number of mononuclear cells harvested. However there were significant differences in the number of PBPC collected on different days. Figure 3 shows the number of GM-CFC collected from patients on days 4–8. Maximum numbers of GM-CFC were collected on day 5 and fewer progenitor cells were harvested after that time. Between 2–3-fold fewer GM-CFC were collected on days 4 ($P < 0.02$), 7 ($P < 0.03$) and 8 ($P < 0.04$) (Student *t*-test). Similar results were obtained for erythroid, eosinophil and multipotential progenitor cells (data not shown). The number of progenitor cells collected by the two collection schedules (days 4, 6, 8 *vs* days 5, 6, 7) was also compared (Figure 3). It was of interest to note that there was no difference in the number of GM-CFC harvested at day 6 using the two collection protocols. Therefore the decline in number of progenitor cells seen with the day 5, 6, 7 collection schedule may reflect a true biological response to G-CSF rather than simply being a consequence of collections on consecutive days. Conversely the day 4, 6, 8 schedule was not associated with heightened levels of progenitor cells that

**Figure 1** Total white cell count (per litre of peripheral blood) and PBPC levels per ml of peripheral blood determined on days 0, 4, 6, and 8 ($n = 8$); (○) or days 0, 5, 6 and 7 ($n = 13$); (●) of G-CSF treatment are presented. These results were obtained from a total of 21 different patients where each point represents mean values from replicate cultures stimulated with either GM-CSF and G-CSF to quantitate non-erythroid colonies (GM-CFC) or with EPO and HPCM to quantitate erythroid colonies.

**Figure 2** Total mononuclear cell count (per litre of leukapheresis sample) and frequency of day 14 CFCs per ml of the leukapheresis product collected on days 0, 4, 6 and 8 ($n = 8$); (○) or days 0, 5, 6 and 7 ($n = 13$); (●) of G-CSF treatment are presented. The results were obtained from a total of 21 different patients where each point represents mean values from replicate cultures stimulated with either GM-CSF and G-CSF to quantitate non-erythroid colonies (GM-CFC) per ml leukapheresis or with EPO and HPCM to quantitate erythroid colonies.

**Table IV** Morphology of colonies from leukapheresis samples

| | *Percentage of non-erythroid colonies* | | | | *Number colonies, per ml* ($\times 10^4$) | |
|---|---|---|---|---|---|---|
| | *M* | *GM* | *Eo* | *Mixed* | *Non-erythroid* | *Erythroid* |
| Baseline | 22 | 54 | 21 | 2 | 0.36 | 0.47 |
| | 12 | 48 | 37 | 3 | 0.055 | 0.22 |
| | 19 | 69 | 12 | 0 | 0.005 | 0.007 |
| Day 5 | 17 | 51 | 23 | 9 | 3.5 | 1.1 |
| | 9 | 51 | 27 | 13 | 2.8 | 4.4 |
| | 12 | 47 | 29 | 12 | 6.4 | 5.2 |

Colonies were examined from cultures stained with Luxol fast blue and haematoxylin and were classified as pure erythroid colonies or non-erythroid colonies (macrophage (M), neutrophil-macrophage (GM), eosinophil (Eo), multipotential (mixed)). A minimum of 90 consecutive colonies were examined except for patient three baseline values (16 colonies). Results are shown prior to receiving G-CSF (Baseline) and after 5 days of G-CSF treatment. The total number of pure-erythroid and non-erythroid colonies per ml of leukapheresis sample is shown.

**Figure 3** Comparison of the total number of non-erythroid colonies (GM-CFC) $ml^{-1}$ of leukapheresis collected on consecutive days (5, 6 and 7, open bars) or alternate days (4, 6 and 8, closed bars). The data is mean ± s.e.m. of results obtained from the 21 patients in Figure 2.

might have been predicted given the 'rest' day between collections.

The total number of GM-CFC harvested using these two schedules was also compared. Harvesting on days 4, 6, 8 produced a total of $1.2 \pm 0.47 \times 10^7$ GM-CFC compared with $2.02 \pm 0.37 \times 10^7$ GM-CFC on days 5, 6, 7. Although because of patient to patient heterogeneity the total number of GM-CFC collected on days 4, 6, 8 was not statistically different from the total number collected on days 5, 6, 7 ($P < 0.2$), seven of the 13 patients on the consecutive day collection schedule achieved levels of GM-CFC $kg^{-1}$ greater than the number required for reconstitution (i.e. $30 \times 10^4$ GM-CFC $kg^{-1}$) (To *et al.*, 1986). In contrast only one of the eight patients on the alternate day collection protocol had greater than $30 \times 10^4$ GM-CFC $kg^{-1}$. This suggests that the preferred schedule for harvesting maximal number of G-CSF mobilised PBPC was days 5, 6, 7.

### *Mobilisation of PBPC after two cycles G-CSF*

In an attempt to maximise PBPC collected by leukapheresis, two patients received two cycles of G-CSF with 10 weeks (Patient 1) or 5 weeks (Patient 2) between cycles. The WCC and number of GM-CFC collected are presented in Table V. For patient 1 there was a poor response to G-CSF in the first cycle with only a 4-fold increase observed in the WCC. A similar low level response was observed after administration of the second cycle of G-CSF. Low levels of GM-CFC were observed in the leukapheresis product after both the first ($7.2 \times 10^4$ GM-CFC $kg^{-1}$) and second ($6.4 \times 10^4$ GM-CFC $kg^{-1}$) cycle of G-CSF. For patient 2, G-CSF increased the WCC by approximately 9-fold and a similar effect was observed with the second cycle of G-CSF therapy. As with patient 1, there was no difference in GM-CFC levels obtained in the leukapheresis product after 1 or 2 cycles of G-CSF. Thus, a prior cycle of G-CSF did not appear to influence subsequent mobilisation of PBPC eithuer positively or negatively and a poor response to the first cycle predicted a poor response to the second cycle of G-CSF.

### *G-CSF mobilises Pre-CFC*

To determine whether an early population of progenitor cells ('pre-CFC') was mobilised by G-CSF and could be detected

**Table V** Progenitor cell levels and white cell count following two cycles G-CSF

| | *Total WCC* ($\times 10^9 l^{-1}$) | | *GM-CFC ml*$^{-1}$ ($\times 10^4$) | | *GM-CFC per Leukapheresis* ($\times 10^4$) | | *GM-CFC kg*$^{-1}$ ($\times 10^4$) | |
|---|---|---|---|---|---|---|---|---|
| *Day* | *Cycle 1* | *Cycle 2* | *Cycle 1* | *Cycle 2* | *Cycle 1* | *Cycle 2* | *Cycle 1* | *Cycle 2* |
| *Patient 1* | | | | | | | | |
| 0 | 4.4 | 2.96 | 0.011 | <0.002 | 2.18 | <0.37 | 0.038 | <0.006 |
| 5 | 16.35 | 19.8 | 0.68 | 0.42 | 135.4 | 83.9 | 2.33 | 1.45 |
| 6 | 16.32 | 15.5 | 1.16 | 0.372 | 233 | 74.4 | 4.01 | 1.28 |
| 7 | 7.0 | 13.9 | 0.24 | 1.06 | 47.9 | 212.2 | 0.83 | 3.66 |
| | | | | | | Total | 7.2 | 6.4 |
| *Patient 2* | | | | | | | | |
| 0 | 2.94 | 3.16 | 0.063 | 0.008 | 12.6 | 1.5 | 0.189 | 0.023 |
| 4 | 19.2 | 21.4 | 3.3 | 3.7 | 660 | 740 | 9.94 | 11.14 |
| 6 | 19.08 | 29.4 | 3.7 | 3.4 | 740 | 680 | 11.1 | 10.24 |
| 8 | 23.7 | 31.3 | 3.4 | 4.9 | 680 | 980 | 10.2 | 14.76 |
| | | | | | | Total | 31.3 | 36.1 |

Progenitor cell data represents mean value obtained from replicate cultures. See Table I for patient details.

in the leukapheresis product, mononuclear cells were cultured in liquid for 7 days then CFC's quantified using a clonal assay as previously described (Moore *et al.*, 1980). The number of CFC generated during the liquid culture phase was compared to the number of input CFC. In the experiments shown in Figure 4, the number of CFC generated was up to 60-fold greater than input CFC. Because greater numbers of CFC were frequently generated after liquid culture compared to the number of input colonies it seemed unlikely that colonies generated after liquid culture simply reflected survival of input progenitor cells. However, as was the case with the progenitor cell levels, the pre-CFC data also showed considerable variation from patient to patient. This is partly illustrated in Figure 4 where one can see the variability in the ratio of input to output colony numbers. Accordingly as a result of treatment with G-CSF, there was an increase in levels of pre-CFC ranging between 15–50-fold above baseline values.

Figure 5 compares the increase in pre-CFC and GM-CFC for samples taken from six patients, on days 5, 6, and 7 after G-CSF treatment. A 15-fold mean increase in pre-CFC

**Figure 4** Comparison of the input number of CFC with number of colonies generated after liquid culture. Adherence depleted mononuclear cells obtained from the leukapheresis product after G-CSF treatment (on either days 5, 6 and 7) were either directly cultured in agar (to determine input CFC) or cultured for 7 days and then assayed in clonal culture ('pre-CFC'). The results presented are from six different experiments where each point represents mean values from replicate cultures stimulated with a combination of GM-CSF, HPCM and EPO.

above baseline was observed on day 5 compared with a 30-fold mean increase in GM-CFC. A similar increase in pre-CFC's was evident on day 6 with a mean increase of 6-fold on day 7. It was interesting to note the apparent dissociation between the fold increase in GM-CFC compared with pre-CFC on day 5 possibly reflecting differences in the kinetics of release in response to G-CSF. These results gave further support for the view that the pre-CFC assay and GM-CFC assay were assessing different populations of cells. This data therefore suggests that G-CSF was able to mobilise earlier populations of cells (pre-CFC) that were also successfully collected by leukapheresis.

### Discussion

This study demonstrated that G-CSF can be used to successfully mobilise progenitor and pre-CFC populations into the blood even in a population of patients heavily pretreated with chemotherapy (Table I). These PBPC were then harvested efficiently using a Fenwal CS-3000 cell separator. The fact that PBPC were efficiently collected using the same machine parameters both before and after G-CSF treatment suggested that the G-CSF mobilised cells shared similar physical properties to PBPC in the resting state. In both situations no CFC were detected in blood returning to the patient. The resting and G-CSF mobilised PBPC also showed similar density characteristics as assessed by behaviour on a Ficoll gradient. As indicatd in Table III virtually 100% of GM-CFC ml$^{-1}$ in the leukapheresis produce were present at the

**Figure 5** Comparison of fold increase above baseline observed for GM-CFC and pre-CFC. Results are mean ± s.e.m. from six different patients for each CFC population. The comparison between GM-CFC and pre-CFC was performed between leukapheresis samples from the same patient.

interface layer for both the resting PBPC and G-CSF mobilised PBPC. This is of particular clinical importance as leukapheresis samples are frequently further processed using a Ficoll gradient to enrich for mononuclear cells prior to transplantation. Our data demonstrate that these techniques can be efficiently used to obtain maximum numbers of G-CSF mobilised progenitor cells.

Following administration of G-CSF, as expected, the peripheral blood white cell count increased by a median of 8-fold above baseline levels. In contrast, the levels of GM-CFC increased by a median of 40 or 60-fold above baseline levels in the peripheral blood or leukapheresis product respectively. This vast difference indicates two events occuring as a result of G-CSF administration. One involves increased numbers of neutrophils via a likely demargination and a later proliferative effect on granulocyte precursors and the other involves the action of G-CSF on a population of progenitor cells leading to the mobilisation of large numbers of CFC into the peripheral blood. As a result, there were increased numbers of GM-CFC, day 14 erythroid progenitors (Figure 1), eosinophil-CFC and multi-CFC (Table IV). This action of G-CSF was not predicted from the known action of G-CSF *in vitro* and is supported by other studies that also demonstrated an increase in megakaryocyte and eosinophil CFC's at G-CSF concentrations as low as $3\ \mu g\ kg^{-1}\ day^{-1}$ (Dührsen *et al.*, 1988). While similar results have been reported for GM-CSF, the number of GM-CFC mobilised PBPC appears at least 10-fold lower than in this current study, but a direct comparison has not been performed. Studies with Multi-CSF (IL-3) showing only a 2-fold increase in PBPC suggests a 'hierarchy' of response in terms of ability to mobilise PBPC. Other hemopoietic regulators may also have important clinical applications in the future. Various animal models (Ulich *et al.*, 1991; Andrews *et al.*, 1991) have been used to study the effect of the recently described kit-ligand or stem cell factor (SCF) *in vivo* (Zsebo *et al.*, 1990). The action of SCF in humans either alone or in combination, however, remains to be determined.

We were also interested in determining whether G-CSF was able to mobilise pre-CFC into the peripheral blood. Murine studies (Nicola & Johnson, 1982) have shown that a population of pre-CFC could be assayed by culturing fractionated fetal liver cells in liquid culture over 7 days in a crude mixture of factors (pokeweed mitogen-stimulated spleen cell-conditioned medium). Similar studies used human bone marrow cells grown in liquid culture in HPCM to assay a population of pre-CFC (Moore *et al.*, 1980) which could then be maintained in long-term culture in the presence of an adherent layer. In this study we also used this technique to assay pre-CFC. In this assay, the number of CFC generated was consistently greater than input CFC, implying that CFC were being generated during the liquid culture phase. In studies using murine fetal liver cells (Nicola & Johnson, 1982), the CFC population died within 5 days while the pre-CFC population reached a maximum at this time. Similar results have been shown for human CFC where the number of GM-CFC fell after 4 days in suspension culture (Jacobsen *et al.*, 1979). It seems likely then that in addition to its effect on mobilising CFC of all lineages G-CSF was also able to mobilise a population of pre-CFC. The increase in this earlier cell population was only up to 50-fold (compared with increases of up to 3000-fold for GM-CFC) and with kinetics suggesting that these cells might be mobilised somewhat later than CFC.

Other studies have utilised cytotoxic drugs with or without CSF's to mobilise PBPC (Gianni *et al.*, 1989; To *et al.*, 1990). Although useful in some situations, such protocols are associated with the side-effects of chemotherapy, are less applicable in patients who have had recent intensive chemotherapy and require administration of drugs that may be relatively inactive in particular malignancies. The protocol described in this study may be more widely applicable because it is not associated with these problems – the only side effects being mild bone pain during G-CSF administration and thrombocytopenia during leukapheresis (requiring platelet transfusion in two patients of 21) (Sheridan *et al.*, 1992). In addition the yield of PBPC using chemotherapy techniques (without addition of CSF's) is 10–100-fold less than with G-CSF alone (Gabrilove *et al.*, 1988; To *et al.*, 1984). However, based on chemotherapy mobilised PBPC a minimum number of $30 \times 10^4$ GM-CFC $kg^{-1}$ is estimated to be required for subsequent engraftment using PBPC transfusion alone (To *et al.*, 1986). When reinfused, chemotherapy mobilised PBPC hasten neutrophil and platelet recovery following high dose chemotherapy. Similar accelerated platelet recovery was observed when the G-CSF mobilised PBPC from this study were reinfused following high dose chemotherapy (Sheridan *et al.*, 1992).

There was considerable variability in responses to G-CSF. This most probably reflects the heterogeneity in this heavily pre-treated group of patients. In addition there was variability for each patient between the period since having last received myelotoxic chemotherapy treatment and entry onto the study (Table I). Thus these patients would be expected to have different hemopoietic stem cell reserves leading to the variable response to G-CSF although intrinsic individual variation might also be expected in response to G-CSF. This issue could be better addressed in patients who had not received prior chemotherapy. Despite this variation, this technique was successful in harvesting $>30 \times 10^4$ GM-CFC $kg^{-1}$ in eight of 21 patients.

Two alterations in the collection protocol were analysed in an attempt to optimise mobilisation and collection of PBPC. One addressed the question of leukapheresis collection schedule. It was possible that harvesting of progenitor cells on consecutive days may have contributed to the decline in PBPC levels between days 5 and 7. Therefore, progenitor cells were subsequently harvested on days 4, 6 and 8. This did not increase the number of PBPC obtained on alternate days and actually resulted in half the total number of PBPC being collected due to lower levels at day 4 and 8. A second approach involved the administration of two cycles of G-CSF. In two patients there was no difference in the ability of G-CSF to mobilise progenitor cells after one or two cycles of G-CSF.

The mechanism involved in G-CSF induced PBPC mobilisation is unknown. Several studies have failed to demonstrate an increase in progenitor cells in bone marrow in response to G-CSF (Dührsen *et al.*, 1988), GM-CSF (Socinski *et al.*, 1988; Haas *et al.*, 1990) or Multi-CSF (IL-3) (Ottoman *et al.*, 1990). In addition the appearance of circulating progenitor cells of all lineages suggests a non-specific mechanism that can be triggered by general insults (such as chemotherapy) as well as by the hemopoietic regulators. However, this mechanism appears not to be totally non-specific as one of the most common subpopulations of bone marrow progenitor cells (day 7 GM-CFC) was not observed in the circulation after G-CSF treatment and furthermore, administration of IL-4 did not mobilise progenitor cells into the peripheral blood (De Luca *et al.*, unpublished results).

In summary, the ability to mobilise and collect PBPC using this non-toxic and efficient procedure has important implications for transplantation and high-dose chemotherapy procedures.

This work was supported in part by grants from the Anti-Cancer Council of Victoria, the National Health and Medical Research Council, Canberra and the Victorian Health Promotion Foundation.

### References

ANDREWS, R.G., KNITTER, G.H., BARTLEMEZ, S.H., LANGLEY, K.E., FARRAR, D., HENDREN, W., APPLEBAUM, F.R., BERNSTEIN, I.D. & ZSEBO, K.M. (1991). Recombinant human stem cell factor, a c-kit ligand, stimulates hematopoieses in primates. *Blood*, **78**, 1975–1980.

BARR, R.D. & MCBRIDE, J.A. (1982). Haemopoietic engraftment with peripheral blood cells in treatment of malignant disease. *Br. J. Haematol.*, **51**, 181–187.

DÜHRSEN, V., VILLEVAL, J.L., BOYD, J., KANNOURAKIS, G., MORSTYN, G. & METCALF, D. (1988). Effects of recombinant human granulocyte colony-stimulating factor on hematopoietic progenitor cells in cancer patients. *Blood*, **72**, 2074–2081.

GABRILOVE, J.L., JAKULSOWSKI, A., FAIN, K., GROUS, J., SCHER, H., STERNBERG, C., YAGODA, A., CLARKSON, B., BONILLA, M.A., OETTGEN, H.F., ALTON, K., BOONE, T., ALTROCK, B., WELTE, K. & SOUZA, L. (1988). Phase I study of granulocyte colony-stimulating factor in patients with transitional cell carcinoma of the urothelium. *J. Clin. Invest.*, **82**, 1454–1461.

GIANNI, A.M., SIENNA, S., BREGNI, M., TARELLA, C., STERN, A.C., PILERI, A. & BONADONNA, G. (1989). Granulocyte-macrophage colony-stimulating factor to harvest circulating haemopoietic stem cells for autotransplantation. *Lancet*, **2**, 580–585.

HAAS, R., HO, A.D., BREDTHAUER, U., CAYEUX, S., EGERER, G., KNAUF, W. & HUNSTEIN, W. (1990). Successful autologous transplantation of blood stem cells mobilized with recombinant human granulocyte-macrophage colony-stimulating factor. *Exp. Hematol.*, **18**, 94–98.

JACOBSEN, N., BROXMEYER, H.E., GROSSBARD, E. & MOORE, M.A.S. (1979). Colony-forming units in diffusion chambers (CFU-d) and colony-forming units in agar culture (CFC-c) obtained from normal human bone marrow: a possible parent-progeny relationship. *Cell Tissue Kinet.*, **12**, 213–226.

METCALF, D. (1984). *Clonal culture of hemopoietic cells: techniques and applications*, pp. 19–92. Elsevier, Amsterdam.

METCALF, D. & NICOLA, N.A. (1983). Proliferative effects of purified granulocyte colony-stimulating factor (G-CSF) on normal mouse hemopoietic cells. *J. Cell. Physiol.*, **116**, 198–206.

MOORE, M.A.S., BROXMEYER, H.E., SHERIDAN, A.P.C., MEYERS, P.A., JACOBSEN, N. & WINCHESTER, R.J. (1980). Continuous human bone marrow culture: Ia antigen characterization of probable pluripotential stem cells. *Blood*, **55**, 682–690.

NAGATA, S., TSUCHIYA, M., ASANO, S., KAZIRO, Y., YAMAZAKI, T., YAMAMOTO, O., HIRATA, Y., KUBOTA, H., OHEDA, M., NOMURA, H. & ONO, M. (1986). Molecular cloning and expression of cDNA for human granulocyte colony-stimulating factor. *Nature*, **319**, 415–418.

NICOLA, N.A. (1990). Granulocyte colony-stimulating factor. In *Colony-Stimulating Factors – Molecular and Cellular Biology*, Dexter, M., Garland, J.M. & Testa, N.G. (ed), p. 77–109. Marcel Dekker Inc: New York and Basel.

NICOLA, N.A. & JOHNSON, G.R. (1982). The production of committed hemopoietic colony-forming cells from multipotential precursor cells *in vitro*. *Blood*, **60**, 1019–1029.

NICOLA, N.A., METCALF, D., JOHNSON, G.R. & BURGESS, A.W. (1979). Separation of functionally distinct human granulocyte-macrophage colony stimulating factors. *Blood*, **54**, 614–627.

OTTOMAN, O.G., GANSER, A., SEIPELT, G., EDER, M., SCHULZ, G. & HOELZER, D. (1990). Effects of recombinant human IL-3 on human hematopoietic progenitor and precursor cells *in vivo*. *Blood*, **76**, 1494–1502.

RICHMAN, C.M., WEINER, R.A. & YANKEE, R.A. (1976). Increase in circulating stem cells following chemotherapy in man. *Blood*, **47**, 1031–1039.

SHERIDAN, W.P., BEGLEY, C.G., JUTTNER, C.A., SZER, J., TO, L.B., MAHER, D., MCGRATH, K.M., MORSTYN, G. & FOX, R.M. (1992). Hemopoietic recovery following infusion of autologous bone marrow and G-CSF mobilized peripheral blood progenitor cells after high dose chemotherapy. *Lancet*, **1**, 640–644.

SHERIDAN, W.P., MORSTYN, G., WOLF, M., DODDS, A., LUSK, J., MAHER, D., LAYTON, J.E., GREEN, M.D., SOUZA, L. & FOX, R.M. (1989). Granulocyte colony-stimulating factor and neutrophil recovery after high-dose chemotherapy and autologous bone marrow transplantation. *Lancet*, **2**, 891–895.

SOCINSKI, M.A., ELIAS, A., SCHNIPPER, L., CANNISTRA, S.A., ANTMAN, K.H. & GRIFFIN, J.D. (1988). Granulocyte-macrophage colony stimulating factor expands the circulating haemopoietic progenitor cell compartment in man. *Lancet*, **1**, 1194–1198.

STIFF, P.J., KOESTER, A.R. & LANZOTTI, V.J. (1986). Autologous transplantation using peripheral blood stem cells. *Exp. Hematol.*, **14**, 465 (abstr).

TO, L.B., DYSON, P.G. & JUTTNER, C.A. (1986). Cell-dose effect in circulating stem-cell autografting. *Lancet*, **2**, 404–405.

TO, L.B., HAYLOCK, D.N., KIMBER, R.J. & JUTTNER, C.A. (1984). High levels of circulating haemopoietic stem cells in very early remission from acute non-lymphoblastic leukemia and their collection and cryopreservation. *Br. J. Haematol.*, **58**, 399–410.

TO, L.B., SHEPPARD, K.M., HAYLOCK, D.N., DYSON, P.G., CHARLES, P., THORP, D.L., DALE, B.M., DART, G.W., ROBERTS, M.M., SAGE, R.E. & JUTTNER, C.A. (1990). Single high doses of cyclophosphamide enable the collection of high numbers of hemopoietic stem cells from the peripheral blood. *Exp. Hematol.*, **18**, 442–447.

ULICH, T.R., DEL CASTILLO, J., MCNEICE, I.K., YI, E.S., ALZONA, C.P., YIN, S. & ZSEBO, K.M. (1991). Stem cell factor in combination with granulocyte colony-stimulating factor (CSF) or granulocyte-macrophage CSF synergistically increases granulopoiesis *in vivo*. *Blood*, **78**, 1954–1962.

VILLEVAL, J.L., DÜHRSEN, V., MORSTYN, G. & METCALF, D. (1990). Effect of recombinant human granulocyte-macrophage colony stimulating factor on progenitor cells in patients with advanced malignancies. *Br. J. Haematol.*, **74**, 36–44.

ZSEBO, K.M., WILLIAMS, D.A., GEISSLER, E.N., BROUDY, V.C., MARTIN, F.H., ATKINS, H.L., HSU, R.Y., BIRKETT, N.C., OKINO, K.H., MURDOCK, D.C., JACOBSEN, F.W., LANGLEY, K.E., SMITH, K.A., TAKEISHI, T., CATTANACH, B.M., GALLI, S.J. & SUGGS, S.V. (1990). Stem cell factor is encoded at the S1 locus of the mouse and is the ligand for the c-kit tyrosine kinase receptor. *Cell*, **63**, 213–224.